# Preoperative nTMS analysis: a sensitive tool to detect imminent motor deficits in brain tumor patients

**DOI:** 10.1007/s00701-024-06308-3

**Published:** 2024-10-21

**Authors:** Ina Moritz, Melina Engelhardt, Tizian Rosenstock, Ulrike Grittner, Oliver Schweizerhof, Rutvik Khakhar, Heike Schneider, Andia Mirbagheri, Anna Zdunczyk, Katharina Faust, Peter Vajkoczy, Thomas Picht

**Affiliations:** 1 Department of Neurosurgery, Charité - Universitätsmedizin, Berlin, Germany, Image Guidance Lab, Luisenstraße 58-60, 10117 Berlin, Germany; 2 Institute of Biometry and Clinical Epidemiology, Charité – Universitätsmedizin Berlin, Charitéplatz 1, 10117 Berlin, Germany; 3 Einstein Center für Neurowissenschaften, Charité – Universitätsmedizin Berlin, Charitéplatz 1, 10117 Berlin, Germany; 4 Berlin Simulation and Training Center, Charité, Charitéplatz 1, 10117 Berlin, Germany; 5 Cluster of Excellence: “Matters of Activity. Image Space Material”, Humboldt Universität Zu Berlin, Unter Den Linden 6, 1099 Berlin, Germany; 6 Berlin Institute of Health (BIH), Translationsforschungsbereich Der Charité-Universitätsmedizin Berlin, Anna-Louisa-Karsch-Str. 2, 10178 Berlin, Germany; 7 Department of Neurosurgery and Center for Spinetherapy, Helios Klinikum Berlin - Buch, Medical School Berlin (MSB), Schwanebecker Chaussee 50, 13125 Berlin, Germany; 8 Department of Plastic and Reconstructive Surgery, Unfallkrankenhaus Berlin, Warener Straße 7, 12683 Berlin, Germany; 9 Department of Neurosurgery, Universitätsklinikum Mannheim, Universitätsmedizin Mannheim, Universität Heidelberg, Theodor-Kutzer-Ufer 1-3, 68167 Mannheim, Germany

**Keywords:** Navigated transcranial magnetic stimulation, Risk stratification model, Brain tumor surgery, Cortical spinal tract, Recruitment curve, Cortical silent period

## Abstract

**Background:**

One of the challenges in surgery of tumors in motor eloquent areas is the individual risk assessment for postoperative motor disorder. Previously a regression model was developed that permits estimation of the risk prior to surgery based on topographical and neurophysiological data derived from investigation with nTMS (navigated Transcranial Magnetic Stimulation). This study aims to analyze the impact of including additional neurophysiological TMS parameters into the established risk stratification model for motor outcome after brain tumor surgery.

**Methods:**

Biometric and clinical data of 170 patients with glioma in motor eloquent areas were collected prospectively. In addition, the following nTMS parameters were collected bihemispherically prior to surgery: resting motor threshold (RMT), recruitment curve (RC), cortical silent period (CSP) and a nTMS based fibertracking to measure the tumor tract distance (TTD). Motor function was quantified by Medical Research Council Scale (MRCS) preoperatively, seven days and three months postoperatively. Association between nTMS parameters and postoperative motor outcome was investigated in bivariate and multivariable analyses.

**Results:**

The bivariate analysis confirmed the association of RMT ratio with the postoperative motor outcome after seven days with higher rates of worsening in patients with RMT ratio > 1.1 compared to patients with RMT ratio ≤ 1.1 (31.6% vs. 15.1%, p = 0.009). Similarly, an association between a pathological CSP ratio and a higher risk of new postoperative motor deficits after seven days was observed (35.3% vs. 16.7% worsening, p = 0.025). A pathological RC Ratio was associated postoperative deterioration of motor function after three months (42.9% vs. 16.2% worsening, p = 0.004). In multiple regression analysis, none of these associations were statistically robust.

**Conclusions:**

The current results suggest that the RC ratio, CSP ratio and RMT ratio individually are sensitive markers associated with the motor outcome 7 days and 3 months after tumor resection in a presumed motor eloquent location. They can therefore supply valuable information during preoperative risk–benefit-balancing. However, underlying neurophysiological mechanisms might be too similar to make the parameters meaningful in a combined model.

## Introduction

A key trade-off in brain tumor surgery is the balance between preservation of functional integrity and maximal resection [9, 32, 33]. Since postoperative functional deficits correlate with a worsened quality of life and also shorter survival times, identification of risk factors for development of such deficits is crucial [15, 21, 24–26, 31]. Individualized preoperative planning based on standardized risk stratification has the potential to minimize postoperative deficits [15, 21, 24–26]. Navigated Transcranial Magnetic Stimulation (nTMS) is a well-established tool for preoperative localization of motor eloquent areas [1, 9, 15, 21, 24, 34, 36, 39, 40]. It has been integrated in preoperative surgical planning to select the best suited surgical strategy for tumor resection [21, 22, 24]. Various studies reported that preoperative nTMS mapping leads to more extensive resections and less functional deficits [14, 21, 23–25, 34]. Further, a regression model for the assessment of the individual risk for the development of a postoperative motor deficits has been established based on data derived from nTMS and tractography. This model is based on three input variables derived from topographical and neurophysiological data acquired through nTMS: 1) the infiltration of the motor cortex by the tumor, 2) the distance of the tumor to the corticospinal tract (CST), and 3) the ratio of the resting motor thresholds (RMT) of both hemispheres, which are used as covariates in the model for the postoperative motor outcome of patients after seven days [25]. In the present study, we assessed cortical silent period (CSP) and recruitment curve (RC) as additional potential predictors as they are easy enough to assess with nTMS in a clinical patient sample [11].

The aim of the present study is to improve the prognostic power of the existing model by including a larger number of patients and additional neurophysiological parameters in patients with glioma in motor eloquent areas to assess the individual risk for a potential postoperative motor disorder.

## Methods

### Patient sample

Prospective data was collected from 170 patients (age range 20–82 years, mean age 52 years, 75 females, 95 males) with malignant glioma in presumed motor eloquent areas. Included patients were evaluated with respect to 1) the anatomical tumor location and possible motor cortex infiltration of the tumor, 2) the distance of the tumor to the cortical spinal tract (TTD) and 3) neurophysiological parameters measured with nTMS to evaluate the motor system’s excitability on the diseased and healthy hemisphere. All patients underwent a preoperative and an early postoperative MRI scan (≤ 24 h). Patients were examined with nTMS and their functional status was assessed with the MRCS and the KPS [4, 30]. The motor status was assessed on POD 7 and after POM 3. Ten patients were lost to POM 3 follow-up, accordingly 160 patients were evaluated for complete follow-up motor status. Further, the following socio-demographical and clinical characteristics were recorded: age at surgery, sex, tumor histology, tumor location and tumor size [6].

#### MRI

Patients underwent a magnetic resonance imaging** (**MRI) protocol with a contrast-enhanced standard 3D magnetization-prepared rapid gradient-echo (MPrage sequence) [17] a fluid-attenuated inversion recovery (FLAIR) sequence, [3, 10] diffusion weighted imaging (DWI) for evaluation of blood brain barrier disorders and for fiber tracking we use a diffusion tensor imaging (DTI) on a Siemens 1.5 or 3 Tesla MRI scanner (Siemens AG, Erlangen, Germany) [25]. The acquired 3D gradient echo sequence was then imported into the nTMS system (NBS 4/5, Nexstim, Helsinki, Finland) and used as a patient-specific navigational dataset. All MR scans were evaluated by experienced neuroradiologists.

#### NTMS

nTMS was applied using a Nexstim NBS 4 or 5 stimulator with a figure of eight coil [1, 24]. Muscle-evoked potentials were recorded from the abductor pollicis brevis (APB) and first dorsal interosseus (FDI) muscles of both hands using disposable silver chloride electrodes (Neuroline 700; Ambu, Ballerup, Denmark). Depending on the tumor location, leg muscles (TA (tibialis anterior), AHB (abductor hallucis brevis)) were additionally recorded. The ground electrode was placed on the left palmar wrist. The exact hotspot for stimulation as well as the optimal coil rotation were defined as the stimulation site, electric-field direction and angulation eliciting the largest muscle evoked responses in the target muscle. The hotspot location as well as optimal rotation and tilting angle were then stored in the system.

### Neurophysiological nTMS parameters

#### RMT

The RMT is a parameter for excitability of the motor system [27, 28]. We started the bihemispheric evaluation of the RMT in October 2007, which included all 170 patients. The RMT was determined as the lowest intensity necessary to evoke a MEP above 50 µV of the FDI muscle in at least 5 out of 10 stimulations. One hundred five percent of the RMT was then used to map the cortical representation of the APB and the FDI muscles bihemispherically. Motor positive stimulation sites (MEPs above 50 µV) over the precentral gyrus were recorded and used as seed regions for the tractography.

#### RC

**Fig. 1:**
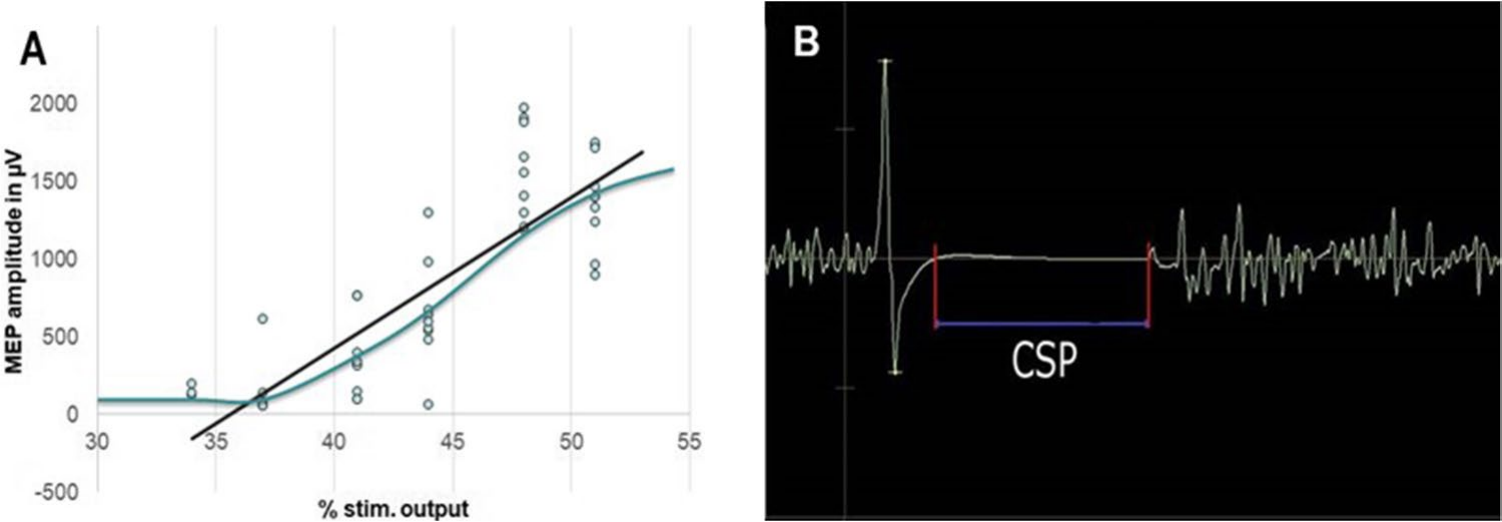
Evaluation of our additional nTMS based parameters. (**A**) For RC, the resulting MEP amplitudes were plotted against the respective stimulation intensities and the slope (blue) of the resulting interpolated linear graph (black) was calculated. (**B**) For CSP, we measured the plateau phase observed in the EMG signal that provided the silent period (latency in ms)

In November 2011 we introduced the RC, which included 124 patients. Briefly, it describes the excitability of the neurons of the corticospinal tract tract [5, 27, 37]. The RC was measured by applying 80 TMS pulses with an intensity between 80 and 140% of the RMT in random order over the FDI hotspot bilaterally. The stimulus interval was 2 s. Thus, this examination required 160 s additionally. The resulting MEP amplitudes were plotted against the respective stimulation intensities and a sigmoidal curve was fitted to the graph. The slope of this graph was then calculated and recorded for analysis, detected by plotting a trend line (Fig. 1A).

#### CSP

In December 2013 we introduced the CSP as a surrogate marker describing the GABA-B receptor-mediated inhibition of cortical excitability, which included 72 patients [12, 29, 30, 39]. CSP was determined by applying 10 stimuli at 130% of the RMT while patients were clenching both fists. Additionally, the ten stimulations of the CSP have a duration of 20 s.

Measurement of the silent period observed in the EMG signal was performed manually post hoc by two independent and experienced researchers (Fig. 1B).

#### Fibertracking

**Fig. 2:**
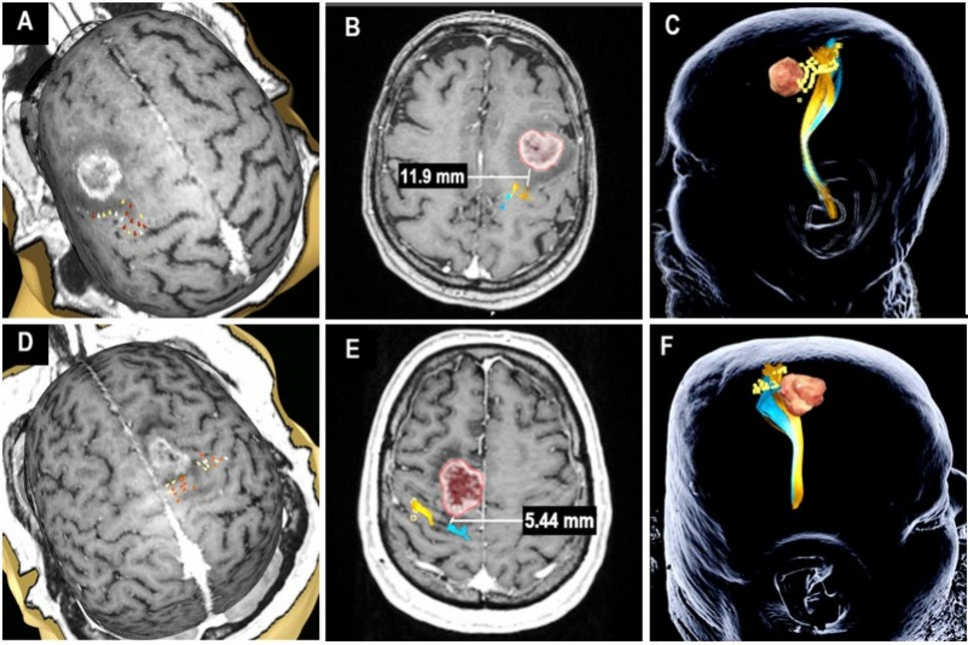
Illustration of nTMS workflow and preoperative planning in an exemplary “low risk” case (**A**-**C**) and a “high risk” case (**D**-**F**). In both cases, nTMS based cortical mapping for the upper and lower (**A**, **D**) extremities was performed. The TMS-based tractography is shown with a TTD > 8 mm (**B**) and a TTD ≤ 8 mm (**E**). In the final preoperative plan for neuronavigation nTMS positive spots are visualized in yellow, upper extremity tracts in orange, lower extremity tracts in blue and the tumor in red (**C**, **F**)

For surgical planning, the locations of positive motor stimulations over the precentral gyrus were exported to the surgical planning software (Brainlab AG, Munich, Germany). The nTMS points were enlarged by 3 mm and used as seed-regions for the fiber tracking of the CST. To optimize the course of the fibers, a second seed-region was placed in the inferior pons [26]. The minimum fiber length was set to 110 mm. Then, the fractional anisotropy (FA) was increased until no more fibers could be tracked and 75% of this upper threshold was used to perform standardized tractography [7, 25, 26]. All tumors were segmented and volumetrically assessed preoperatively [25, 26]. The minimal distance between the 3D calculated fiber tracts and tumor volume was measured manually and recorded for analysis (Fig. 2) [25, 26].

The following information was then provided to the operating neurosurgeons: the extent of infiltration of the motor cortex, the minimum TTD to the CST and the excitability balance between both hemispheres, defined as RMT ratio [15, 21, 25, 26].

### Surgical workflow

The decision on the intraoperative use of nTMS and tractography, which were available in neuronavigation, and the use of intraoperative neuromonitoring and mapping (IONM) was left to the operating surgeon’s discretion [13]. For cortical and subcortical mapping, monopolar, anodic trains of 5 square pulses (0.3 ms, 400 Hz**)** were used. The stimulation intensity was increased in 1 mA steps [26]. For cortical mapping, an upper limit of 20 mA and, for subcortical mapping, of 10 mA was used. Motor function was recorded using subdermal needle electrodes in the FDI, APB, biceps, brachioradialis muscles, the TA and AHB for the representation of the upper and lower extremities. Sustained MEP amplitude reduction of more than 50% and reproducible elicitation of MEPs at 5 mA during subcortical stimulation were usually defined as a stop signal for subcortical resection (5, 7, 32]. Postoperatively, an MRI was performed to measure the extent of resection**.**

### Statistical approach

All nTMS parameters were analyzed for both hemispheres separately as well as by computing the hemispheric ratio [15, 24]. For calculation of this ratio, the hemisphere with the higher value was divided by the hemisphere with the lower value resulting in scores of 1 or greater. This approach was chosen based on a previous study [25], showing that uneven excitability between both hemispheres is associated with a worsened motor outcome. Further, resulting ratios were dichotomized to reflect a normal or abnormal ratio.

**Fig. 3:**
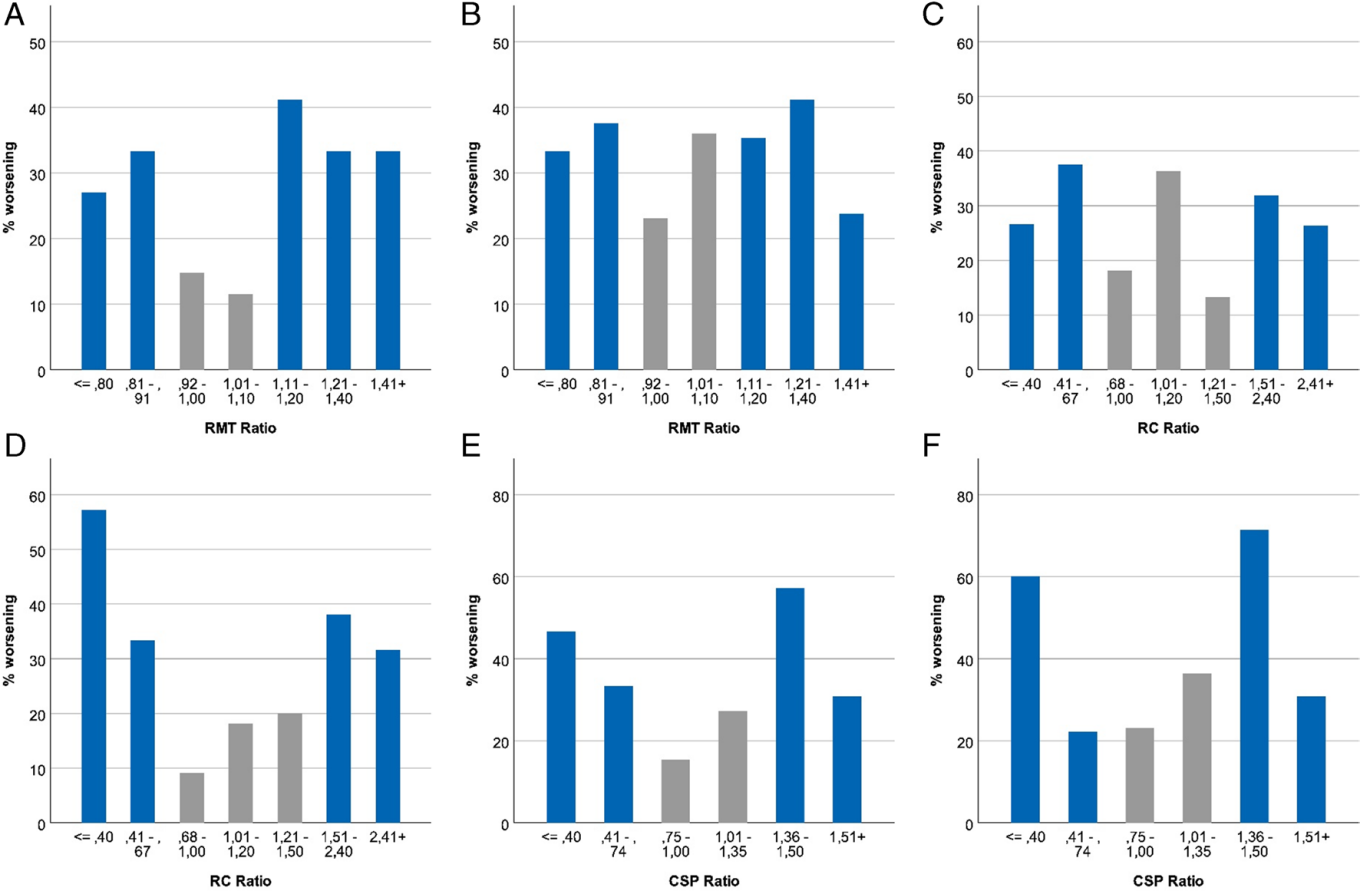
The RMT/RC/CSP ratio of the sick and healthy hemispheric value. (**A**) shows relative frequency of worsening of motor status at 7 days compared to baseline by RMT ratio (≤ 0.8: n = 37, 0.81–0.91: n = 24, 0.92–1.00: n = 27, 1.01–1.10: n = 26, 1.11–1.20: n = 17, 1.21–1.40: n = 18, 1.41 + : n = 21). (**B**) relative frequency of worsening of motor status at 3 months compared to baseline by RMT ratio (≤ 0.8: n = 36, 0.81–0.91: n = 24, 0.92–1.00: n = 26, 1.01–1.10: n = 25, 1.11–1.20: n = 17, 1.21–1.40: n = 17, 1.41 + : n = 21). (**C**) shows relative frequency of worsening of motor status at 7 days compared to baseline by RC ratio (≤ 0.4: n = 15, 0.41–0.67: n = 16, 0.68–1.00: n = 11, 1.01–1.20: n = 11, 1.21–1.50: n = 15, 1.51–2.40: n = 22, 2.41 + : n = 19). (**D**) shows relative frequency of worsening of motor status at 3 months compared to baseline by RC ratio (≤ 0.4: n = 14, 0.41–0.67: n = 15, 0.68–1.00: n = 11, 1.01–1.20: n = 11, 1.21–1.50: n = 15, 1.51–2.40: n = 21, 2.41 + : n = 19). (**E**) shows relative frequency of worsening of motor status at 7 days compared to baseline by CSP ratio (≤ 0.4: n = 15, 0.41–0.74: n = 9, 0.75–1.00: n = 13, 1.01–1.35: n = 11, 1.36–1.50: n = 7, 1.51 + : n = 13). (**F**) shows relative frequency of worsening of motor status at 3 months compared to baseline by CSP ratio (≤ 0.4: n = 15, 0.41–0.74: n = 9, 0.75–1.00: n = 13, 1.01–1.35: n = 11, 1.36–1.50: n = 7, 1.51 + : n = 13)

We have divided the crude RMT/RC/CSP ratio of the sick divided by the healthy hemispheric value. (Fig. 3). Herewith we show that not only a higher value on the diseased compared to the healthy hemisphere, but also conversely a higher value on the healthy compared to the diseased hemisphere is unfavorable.

To distinguish between pathological and non-pathological values of RMT, RC and CSP ratios we used the following cut offs for non-pathological measures: RMT ratio ≤ 1.10, RC ratio ≤ 1.50, CSP ratio ≤ 1.35.

**Fig. 4:**
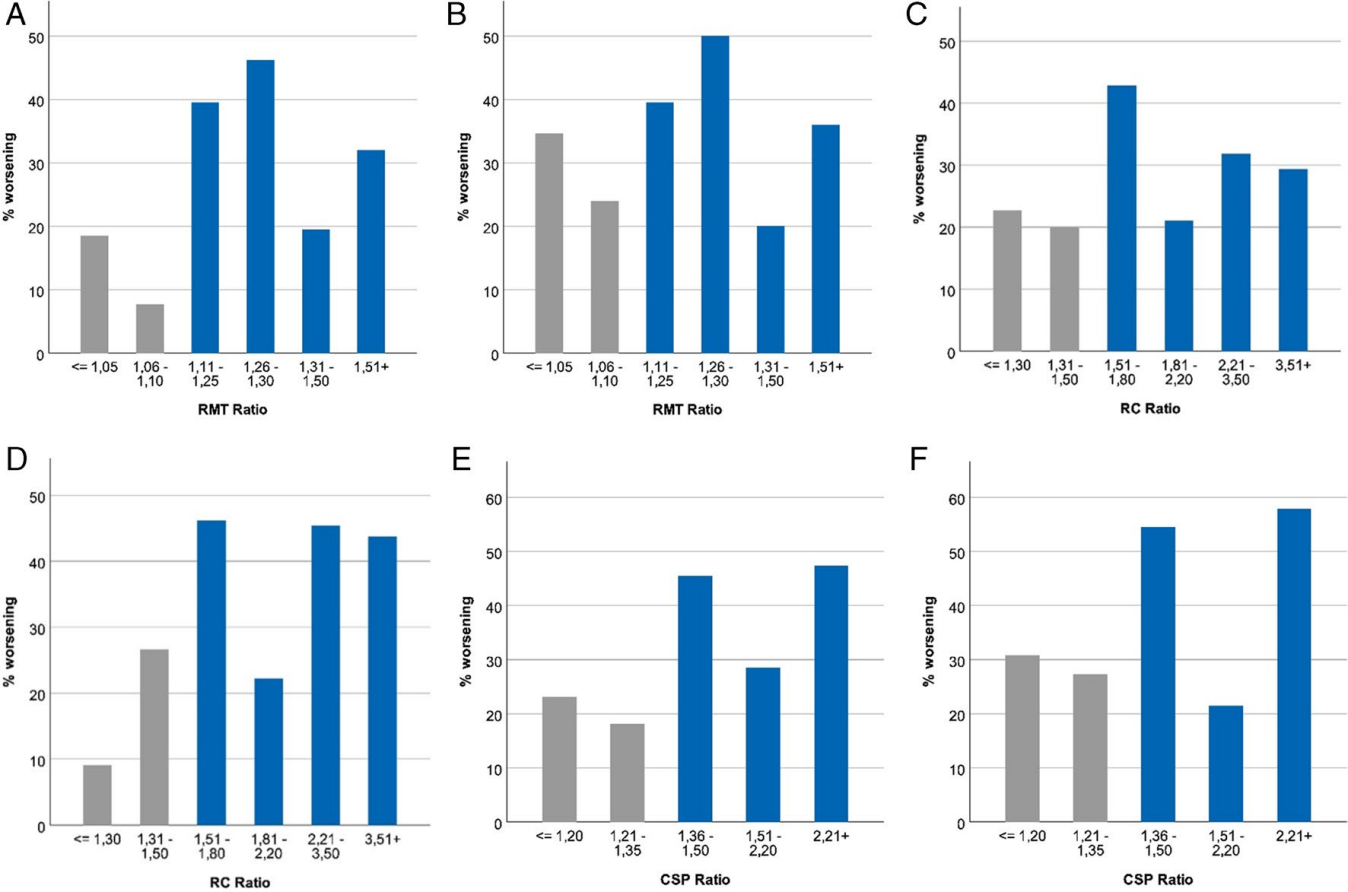
Description regarding the definition of RMT/RC/CSP thresholds. (**A**) presents relative frequency of worsening of motor status at 7 days compared to baseline by RMT ratio (≤ 1.05: n = 27, 1.06–1.10: n = 26, 1.11–1.25: n = 43, 1.26–1.30: n = 13, 1.31–1.50: n = 36, 1.51 + : n = 25). (**B**) presents relative frequency of worsening of motor status at 3 months compared to baseline by RMT ratio (≤ 1.05: n = 26, 1.06–1.10: n = 25, 1.11–1.25: n = 43, 1.26–1.30: n = 12, 1.31–1.50: n = 35, 1.51 + : n = 25). (**C**) presents relative frequency of worsening of motor status at day 7 compared to baseline by RC ratio (≤ 1.3: n = 22, 1.31–1.50: n = 15, 1.51–1.80: n = 14, 1.81–2.20: n = 19, 2.21–3.50: n = 22, 3.51 + : n = 17). (**D**) presents relative frequency of worsening of motor status at 3 months compared to baseline by RC ratio (≤ 1.3: n = 22, 1.31–1.50: n = 15, 1.51–1.80: n = 13, 1.81–2.20: n = 18, 2.21–3.50: n = 22, 3.51 + : n = 16). (**E**) presents relative frequency of worsening of motor status at 7 days compared to baseline by CSP ratio (≤ 1.2: n = 13, 1.21–1.35: n = 11, 1.36–1.50: n = 11, 1.51–2.20: n = 14, 2.21 + : n = 19). (**F**) presents relative frequency of worsening of motor status at 3 months compared to baseline by CSP ratio (≤ 1.2: n = 13, 1.21–1.35: n = 11, 1.36–1.50: n = 11, 1.51–2.20: n = 14, 2.21 + : n = 19)

The ratio thresholds were defined in a data-driven approach. In graphs we illustrate the relation between the particular ratios and the probability of worsening of the motor status by using arbitrary categorizations to get small groups (Fig. 4).

The cut-off value for the RMT was chosen based on a previous publication, showing a range of 90% to 110% of the RMT ratio is associated with an increased risk for a new postoperative motor deficit. We then determined cut-off values for the RC ratio and the CSP ratio in a similar fashion [25].

As descriptive measures we used median values with interquartile range (IQR) if the data were not sufficiently normally distributed. For normally distributed data, we used the mean value with standard deviation (SD).

In the bivariate analyzes, we used the Fisher´s Exact, Mann Whitney U-, Kruskall-Wallis—and Mantel–Haenszel-Test (Linear-by-linear association test) to assess the association of different characteristics with the postoperative motor status or change in motor status. A two-sided significance level of 0.05 was used. No adjustment for multiple testing was applied in this exploratory study. Data analysis was performed using SPSS (IBM SPSS Statistics) and STATA 13 (IC) [25].

### Regression analysis

Variables with a substantial association with change in motor status in the bivariate analyses were included in three different regression models. General ordinal regression using odds ratios (OR) to predict a change in the motor status POD 7 and POM 3. An OR higher than 1 corresponds to a higher probability for postoperative motor function deterioration; an OR less than 1 reflects a lower probability for deterioration. First, the previously postulated model based on the RMT ratio was evaluated in a larger number of patients. (Rosenstock et al., 2016). Secondly, the RC and CSP ratios were added to the model. Of note, not all patients had complete datasets with all measures since bihemispheric measurement of RC and CSP was only introduced during the course of the prospective case collection.

In the third variant, we evaluated the existing model including the aforementioned parameters specifically for patients with a TTD of equal to or less than 8 mm. Based on the already postulated model, this subgroup has been identified as being specifically at risk for development of postoperative deficits [25].

## Results

### Preoperative patient characteristics

In total, 170 patients were included in the analysis. A mean KPS score of 90% (range: 40%—100%) was recorded. The histology showed 143 patients with HHG (high grade glioma) and 27 with LGG (low grade glioma). Tumor location was equally distributed between right (49%) and left (51%) hemisphere. The majority of the tumors was infiltrating the precentral gyrus (33%), followed by tumors that infiltrate the internal capsule (26%) and the premotor and supplementary motor cortices (25%). 16% of all tumors were outside the previously named tumor sites. 100 patients (59%) presented with a MRCS of 5, 54 (32%) with a MRCS of 4 and 16 (9%) with a MRCS of ≤ 3.

### Postoperative status

Preoperative motor status was strongly positively associated with postoperative motor status at both time points 7 days (p = 0.001) and 3 months (p = 0.002) after surgery (median in preoperative group MRCS ≤ 3: post value 3 (IQR: 0.25 – 3.0), in MRCS 4: 4 (IQR: 4.0 – 4.0), in MRCS 5: 5 (IQR: 4.0 – 5.0), Table 1). Patients with a higher pre-operative KPS had a better postoperative motor outcome compared to patients with lower preoperative KPS [(median: 5 (IQR: 4.0 – 5.0) vs. 4 (IQR: 2.0 – 5.0) at 7 days (p = 0.003) and 5 (IQR: 4.0 – 5.0) vs. 4 (IQR: 2.0 – 5.0) at 3 months (p = 0.002)]. A longer symptom duration was associated with worse postoperative motor function at POD 7 and POM 3 with median of 4 (IQR: 4.0 – 5.0) and 3 (IQR: 4.0 –5.0) in patients with symptoms duration of more than 12 weeks compared to median 5 at POD 7 (IQR: 2.0 – 4.0) and POM 3 (IQR: 2.0 – 4.0) in patients with no symptoms (each p < 0.001). Patients of younger age showed a better motor outcome than patients with older age three months postoperatively (median for age ≤ 45: 5 (IQR: 4.0 – 5.0), all other age groups: 4 (IQR: 3.0 – 5.0), p = 0.031). No substantial differences in the motor status were present between high- and low-grade glioma after POD 7 or POM 3. Similarly, for gender and affected hemisphere), no substantial difference in terms of post-operative outcome was found. All patient characteristics and their association with the postoperative motor outcome are presented in Fig. 3a, b and Supplements: Fig. {1a, 1b, 1c} and Table 1).

**Table 1:**
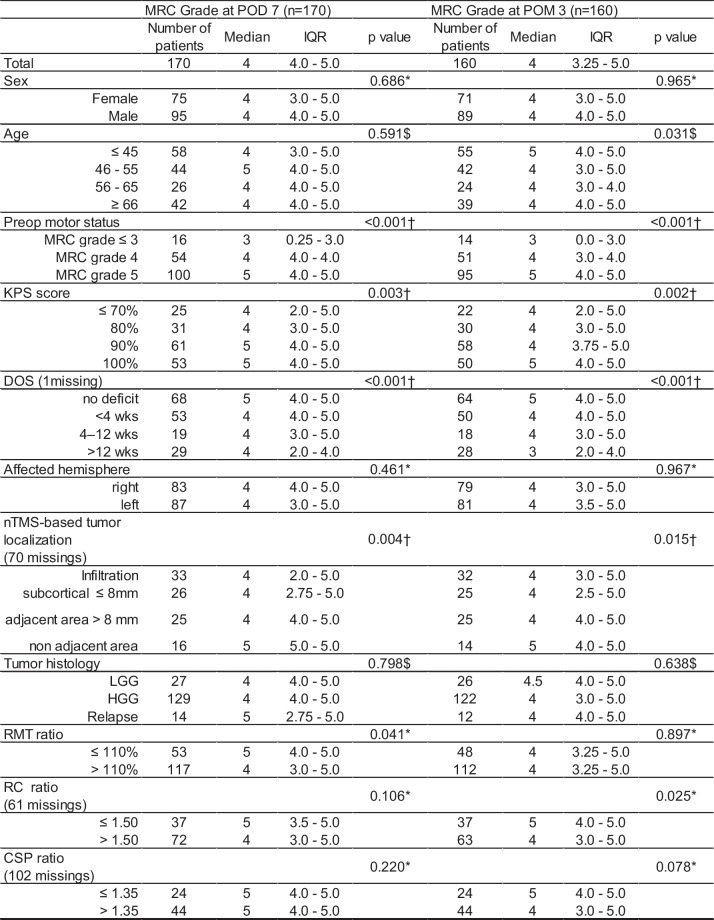
Univariate analysis of motor outcome at POD 7 and POM 3 according to preoperative nTMS variables

*DOS* duration of motor symptoms; *HGG* high-grade glioma; *IQR* interquartile range; *LGG* lowgrade, *HGG* highgrade glioma; RMT -, RC-, CSP ratio = ratio of RMT, RC, CSP dichotomization of affected hemisphere and healthy hemisphere

^*^ Mann–Whitney U-test

^†^ Linear trend test (using Monte Carlo simulations for precision)

^§^ Based on the number of patients with age and tumor histology: 170 patients at POD 7 after surgery and 160 patients at POM 3 after surgery (160 patients were evaluated for follow-up motor status)^27^

### Motor status change

**Fig. 5:**
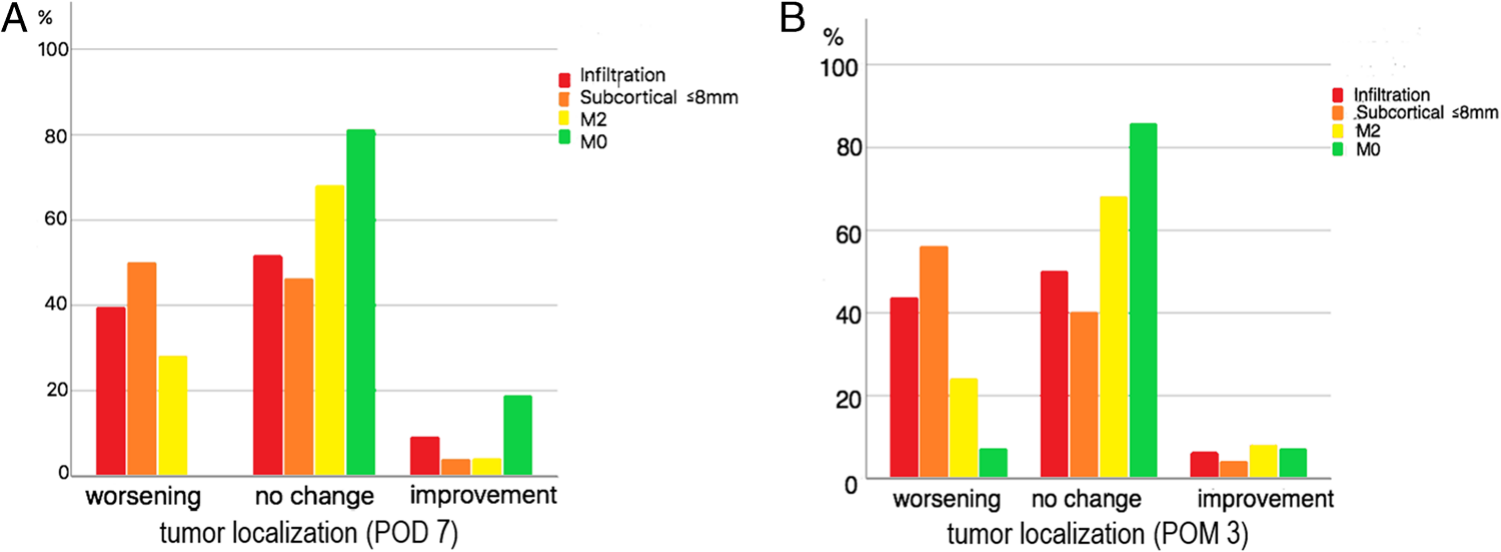
Bar charts comparing the number of patients with improved, unchanged, and worse postoperative motor status after POD 7 (170 patients) and after POM 3 (160 patients), according to the preoperative motor status and according to the distance between the nTMS-based fiber tracts and suspected tumor tissue. Tumor localization: Infiltration (red) = TTD = 0 mm, infiltration of the primary motor cortex; Subcortical (orange) = TTD ≤ 8 mm; M2 (yellow) = TTD > 8 mm, tumor in areas frontally adjacent to the primary motor cortex. M0 (green) = TTD > 8 mm, not adjacent to the primary motor cortex

On POD 7 (n = 170), 45 (26%) of the patients showed a worsening of the motor status, 114 (67%) patients did not show any change and 11 (7.0%) of the patients improved. After POM 3 (n = 160 (10 patients were lost to 3-month follow-up)), a total of 55 (34.5%) patients showed a worsening of motor function compared to before surgery, 95 (59.5%) presented without any motor change and 10 (6.0%) showed a motor improvement (Fig. 5a, b). In this regard a relevant change of motor status could be found after POD7 (p < 0.0001), and after POM3 (p = 0.001).

#### RMT

Initially, we evaluated the association of the nTMS parameters ratio with a worsening of the postoperative motor status in a bivariate analysis after POD7 and POM3.

The RMT was recorded in 170 patients with an average value of 72.9 (SD 23.8) V/m in the diseased hemisphere and 70.9 (SD 18.6) V/m in the healthy hemisphere. The median RMT ratio was 1.19 (IQR 1.09–1.38).

**Fig. 6:**
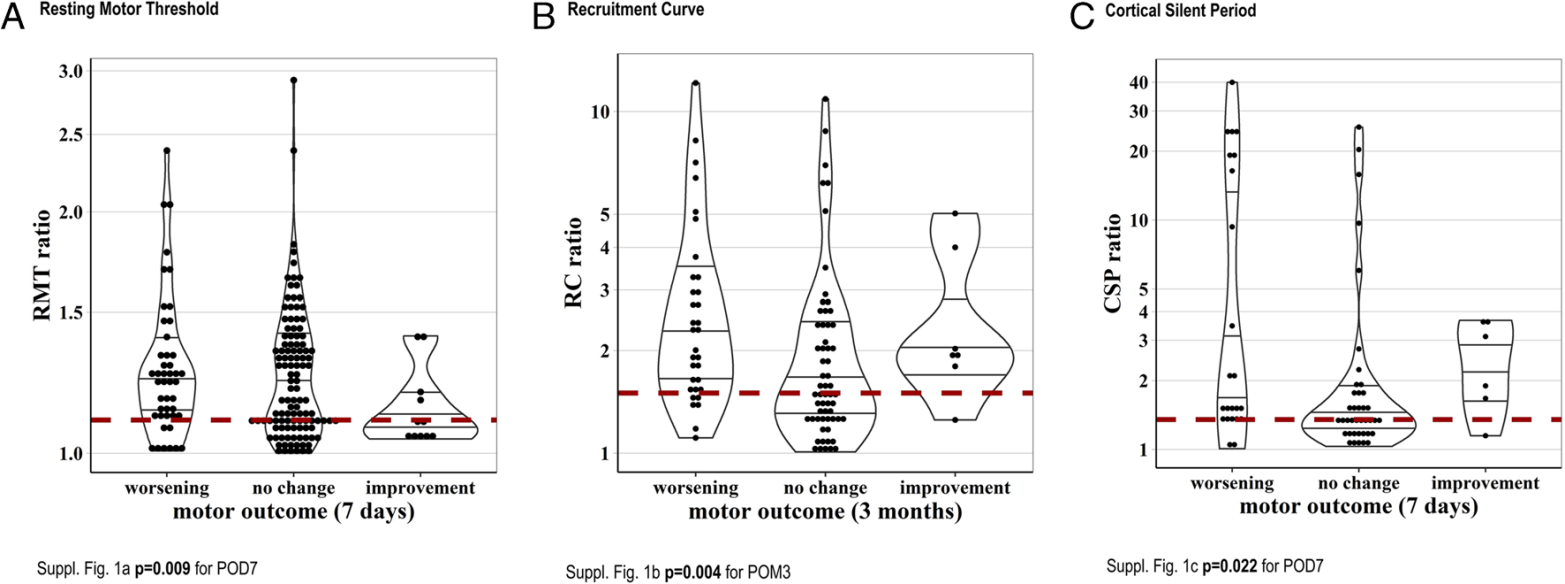
**a-c** shows the RMT, RC and CSP ratio presented in violinplots according the postoperative motor outcome. The continuous line shows the reference range; the first line: the 25th percentile; second line: the median, third line: the 75th Percentile

A pathological RMT ratio was associated with a higher risk of worsening of motor function seven days postoperatively (31.6% vs. 15.1%) and a lower probability of improvement of motor status (6.5% vs. 13.2%, p = 0.009). With regard to changes from preoperative status to 3 months after surgery, almost no difference was present between patients with pathological and normal RMT ratio (worsening: 34.0% vs. 35.4%, improvement 10.0% vs. 10.4%, p = 0.326) (Fig. 6a).

#### RC

The RC was measured in 124 patients on the affected hemisphere with a median of 157 (IQR: 91–263) µV/% and in 115 patients on the healthy hemisphere with a median of 128 (IQR: 77–218) µV/%. The RC ratio could be investigated in 109 patients (Fig. 4). The median RC ratio was 1.88 (IQR: 1.39–2.71).

In a bivariate analysis a pathological RC ratio was associated with a higher risk of worsening of motor function after POM 3 (42.9% vs. 16.2% worsening, p = 0.004) (Fig. 6b). It was further associated with a worse preoperative motor function (p = 0.019). However, proportions of worsening at POD 7 were similar in both groups (30.6% vs. 21.6% worsening, p = 0.343).

#### CSP

The CSP was recorded in 72 patients. The affected hemisphere showed an average CSP duration of 112 (SD: 71) ms and the healthy hemisphere an average of 129 (SD: 54) ms.

However, two patients showed in the diseased hemisphere a latency of 0 ms (both were unable to tense their muscles due to a paralysis (MRCS = 0), making the calculation of a CSP ratio impossible. In one patient the CSP could only be determined on the affected, in another only on the healthy hemisphere. Hence the CSP ratio could be investigated in 68 patients.

In a bivariate analysis, an abnormal CSP ratio was associated with a postoperative deterioration of motor function after seven days (43.2% vs. 16.7% worsening, p = 0.022) (Fig. 6c). However, this effect was less pronounced 3 months after surgery (43.2% vs. 25.0% worsening, p = 0.115).

#### Fibertracking

100 patients received a tractography preoperatively. 41 (41%) patients showed a (TTD) greater than 8 mm and 59% (59%) patients less or equal 8 mm.

In patients with a TTD of less or equal than 8 mm, the probability of a worsening of motor function seven days (43.9% versus 11.8% p = 0.005) and three months after tumor resection (47.7% versus 12.9% p = 0.001) was higher than in other patients (Fig. 5).

Patients with tumor infiltration of the precentral gyrus and a TTD ≤ 8 mm showed a higher risk of having a postoperative motor deterioration after POD 7 and POM 3 (39–56% patients with worsening vs. TTD > 8 mm in adjacent and not adjacent areas of the motor cortex: 0–28% patients with worsening).

### Ordinal regression analysis

Firstly, the association of the preoperative motor status, RMT ratio and tumor localization with a postoperative change in motor function after 7 days and 3 months was evaluated). After adjustment for preoperative motor status and tumor localization, a pathological RMT ratio was not substantially associated with a change in motor function after POD 7 (OR: 2.14, 95% CI: 0.85–5.45, p = 0.108) or the third month (OR: 1.33, 95%CI: 0.54–3.28, p = 0.541). Model fit showed low values of variance explanation with R^2^ = 9% for POD 7 and 8% for POM 3. Further, we added a subgroup analysis of 67 patients at POD 7 and 64 patients at POM 3 including only cases (patients) with full data availability to confirm our key findings.

(Table 2).

**Table 2:**
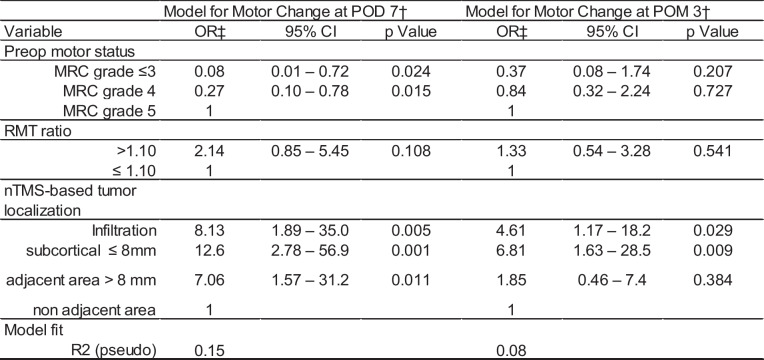
Ordinal regression analysis: RMT ratio

^†^ Analysis based on 100 patients at POD 7 and 96 patients at POM 3

^‡^ An OR higher than 1 stands for a higher probability of deterioration in the preoperative motor status.^27^

Parameters improved the model for 3 months (R^2^ = 15%), demonstrating higher risks of worsening of motor status for those patients with higher RC or CSP ratios (RC OR: 2.50, 95% CI: 0.63–9.81, p = 0.192, CSP OR: 3.67, 95% CI: 0.89–15.30, p = 0.073). The model for POD 7 showed a very low model fit with variance explanation of only 5% (Table 2).

Additionally, we estimated a model for a high-risk subgroup of patients with tumor distance lower than or equal than 8 mm. A pathological RMT Ratio was associated with a higher likelihood of worsening of postoperative motor status after seven days (OR: 2.94, 95% CI: 0.99 – 8.70, p = 0.05) in this sub group. However, this association was weaker for the motor status at third month after surgery (OR: 1.37, 95% CI: 0.48 – 3.94, p = 0.56) (Table 3). Adding the RC ratio to the regression model for the high-risk subgroup, the RMT ratio was again positively associated with worsening in postoperative motor status after seven days (RMT ratio: OR: 3.9, 95% CI: 1.05 – 14.22, p = 0.04), but not three months (OR: 0.97, 95%, CI: 0.27 – 3.44, p = 0.96).

**Table 3:**
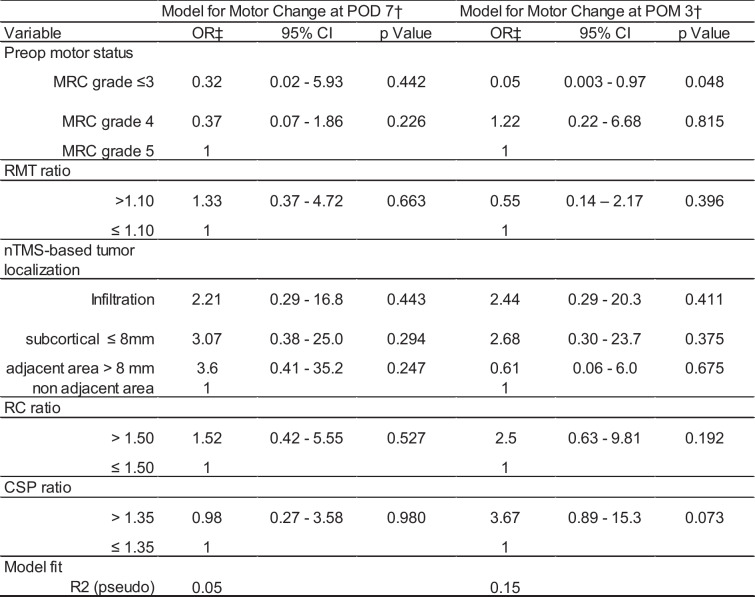
Ordinal regression analysis: the RC ratio and CSP ratio were added to the models

^†^ Analysis based on 67 patients at POD 7 and 64 patients at POM 3

^‡^ An OR higher than 1 stands for a higher probability of deterioration in the preoperative motor status

When adding the CSP ratio to the regression model for the high-risk subgroup, none of the evaluated variables showed a substantial association with the postoperative motor change (RMT ratio: OR: 2.45, 95%, CI: 0.53 – 11.39, p = 0.26; RC ratio: OR: 1.65, 95%, CI: 0.37 – 7.31, p = 0.5; CSP ratio: OR: 1.07, 95%, CI: 0.26 – 4.43, p = 0.98,) or POM 3 (RMT ratio: OR: 0.47, 95%, CI: 0.99 – 2.24, p = 0.34; RC ratio: OR: 1.99, 95%, CI: 0.46 – 8.5, p = 0.36; CSP ratio: OR: 3.67, 95%, CI: 0.81 – 16.7, p = 0.09).

## Discussion

### Main Finding

The development of exploratory models for predicting functional impairment is an ongoing challenge in medicine. The aim of the present study was the validation and improvement of an established model predicting the postoperative motor outcome in brain tumor patients.

We confirmed that nTMS-based neurophysiological parameters individually indicate the risk for development of postoperative motor deficits. The bivariate analysis of all examined preoperative nTMS parameters showed some association of these measures with the postoperative motor outcome. Specifically, in patients with a new postoperative motor deficit after seven days, an abnormal RMT ratio and a pathological CSP ratio preoperatively was observed. Patients with a worsening of motor function after three months showed impaired RC ratios. However, against our initial hypothesis their combination in a combined predictive model was not advantageous, as shown by the regression analysis.

Only for a specifically endangered subgroup with a TTD less or equal than 8 mm, an association of pathological RMT ratio with a worsening of the postoperative motor outcome of patients after seven days was found after adjustment for preoperative motor status [7, 8, 25, 26].

### Association of nTMS parameters with changes in motor function

Despite finding associations, overall model fit was low (below 20% of variance explanation). In the bivariate analysis, we were able to replicate some associations of the risk stratification model proposed in an earlier study [25] in a larger patient sample, e.g., the association of the distance of the tumor to the CST and the RMT ratio with the postoperative motor deterioration after seven days.

Further, association of other nTMS parameters (RC and CSP) with changes in motor outcome could be demonstrated in this study. Using categorizations of the RC ratio, motor changes were shown at POD 7 and POM 3 (Figs. 1 and 2): Especially at POM 3, patients with RC ratios > 1.5 have more worsening of their motor status compared to those with an RC ratio ≤ 1.5. Similarly, patients with a CSP ratio > 1.35 have a higher probability of motor worsening than patients with a lower CSP ratio ≤ 1.35. Yet, these effects are not very pronounced. Since this is a study which explores potential associations, it is possible that with a larger population more suitable cut off values could be defined. Insofar these cut offs reflect a pragmatic approach to illustrate if there are associations of RC and CSP ratio to motor status changes at all. Concrete impacts on surgical decision making may be studied in the future, yet with the awareness that surgical decision‐making strategies are highly complex and influenced by many factors such as tumor entity, age and personal life situation of the patient, location and tumor tract distance (TTD).

However, we failed to show an advantage of an extended risk-stratification model containing additional TMS (RC and CSP ratio) parameters. One possible explanation for this result is a lack in statistical power as only 68 patients provided all required values. While the total sample size was sufficient to detect substantial effects for the bivariate analysis of the RMT ratio, the reduced sample size could have been too small to detect further effects. Not all patients were assessed with the RC or CSP, since these measurements were integrated into clinical practice step-wise over time (nTMS-based DTI tumor localization (70 DTIs missing), RC measurement and RC ratio (61 missing), CSP measurement and CSP ratio (102 missing). Bihemispheric RC and CSP measurements were introduced at a later date, as well as the DTI for nTMS based tumor localization). While the RMT is a crucial measure for assessment of a reliable motor area, other parameters were initially not standard measures for preoperative planning.

We acknowledge that these omissions are a potential source of bias. However, the missing measurements occurred due to changes in the clinical routine; and are therefore completely random (MCAR), which does not bias the results as such despite the lower power in the analysis of these end points.

Another possible explanation for the lack of model improvements after inclusion of several parameters is the similarity of the included nTMS parameters. Determination of the RC and CSP rely on the RMT as their stimulation intensity is defined in relation to the RMT. Further, the RMT and RC are both measures for cortical excitability of the underlying neuronal populations [37, 38]. While the RC can be seen as a more elaborate measurement, both measures have a strong overlap in their underlying neurophysiological mechanisms [19, 20]. They might thus be too similar to show an advantage in model fit when added to the same model. The CSP is a measure for GABAergic inhibition of stimulated neurons [2, 30]. While inhibition is a fundamentally different physiological process compared to excitation, activity of inhibitory neurons influences activity of excitatory neurons [2, 18, 33, 35]. Therefore, also this parameter is physiologically connected to the RMT and RC and does not explain additional variance in the combined analysis.

Interestingly, the RC was identified as a marker for long-term motor impairments, while the RMT and CSP seem to indicate short-term deficits. The RC provides a finer look regarding the excitability at different stimulation intensities and is based on the RMT. The slope of the RC curve indicates the strength and spatial distribution of the corticospinal excitability of the neurons [5, 19, 32, 37]. Preoperatively, the affected hemisphere shows an increased RC in contrast to the healthy hemisphere. With respect to the motor function, we found an increased excitability shown in a steeper curve in patients with a motor deficit compared to patients with a normal muscle strength preoperatively. Patients with a severe motor deficit MRC: < 3 showed an increased mean RC on the diseased hemisphere (195) than patients without a motor deficit (179).

In particular, patients with a postoperative motor deficit after POM 3 have shown an increased RC ratio. Some other studies [34] postulate that patients with stroke have a reduced RC excitability at the axonal level. It has been shown that output and amplification of the RC, especially in higher motor cortices, play an important role [32, 37]. In brain tumor patients, increased neuronal excitability, as evidenced by an increased RC in the affected hemisphere, appears to detect ongoing neuromodulation to maintain motor function in the presence of a growing brain tumor at a different scale than the RMT. If RC disruption occurs, this could reflect more extensive alterations in neuronal signaling than RMT alterations and therefore be a marker for the development of longer-lasting deficits. Thus, in particular, an abnormal preoperative RC ratio could be a valuable index for detailed preoperative planning.

In the analysis of the "tractography-based high-risk group" alone (TTD m ≤ 8 mm), a pathologic RMT ratio was associated with postoperative worsening of motor function at POD 7, allowing further stratification of risk in patients with tumors in close proximity to the corticospinal tract. The RC ratio and CSP ratio showed no association to the postoperative motor deterioration in the high-risk group model. Again, this might be influenced by the smaller sample size when including all parameters.

A progressing pathology is associated with a subsequent change in the motor network and consequently an unequal excitability between both hemispheres, visible in an abnormal RMT ratio [10]. These hemispheric changes might then be strengthened by a strong imbalance in interhemispheric inhibition as it has been reported with the help of nTMS in patients with paresis in stroke research [16, 20]. An increased inhibition of the affected hemisphere by the unaffected hemisphere worsens motor deficits caused by the lesion itself and thus reduces the capacity for functional rehabilitation [10, 25, 37]. Our analysis supports these findings by showing that within a high-risk group an unequal excitability between both hemispheres is a risk factor for postoperative motor deficits. Other parameters such as the RC and CSP are more network-related and might therefore be compensated longer by support of peripheral networks away from the tumor [37].

### NTMS for neurosurgical practice

Of all investigated nTMS parameters, the RMT is the most established and routinely used parameter in preoperative planning [22, 25, 37]. Further, its measurement does not require further offline analysis and is a prerequisite for assessment of the RC and CSP. We acknowledge that the collection of the two extra parameters (RC, CSP) prolongs the measurement and thus, potentially, puts an extra burden on the patient. Still, the examination including measurement of the two additional parameters takes only five minutes longer than the regular examination and none of the patients quit the examination prematurely.

If time for preoperative planning is limited, we recommend relying on the RMT and the anatomical parameters for risk assessment. We then recommend the use of the additional nTMS parameters in one of the following cases: (i) There is evidence for an abnormal interhemispheric inhibition, for example due to an abnormal RMT ratio. In this case, the CSP might add additional information. (ii) There is evidence for a reduced axonal integrity, for example visible in FA values of the CST. In this case, the RC might add additional information (iii) If the TTD is suspected to be lower than or equal to 8 mm, thus classifying the patient at high-risk for a postoperative motor disorder, we recommend measuring all three nTMS parameters. In this case, a detailed preoperative planning can be crucial and special care should be taken during surgery. (iv) Finally, RMT and CSP showed associations specifically for deficits after seven days, while RC was more sensitive to deficits after 3 months. Consequently, these time-considerations can be taken into account as well.

### Limitations

The ratios of the nTMS parameters are based on a data-driven approach and.

were determined arbitrarily.

While the RMT and RC are objectively accessible measures, the CSP is dependent on the patient’s ability to clench his fists. The EMG quality is an important factor for all parameters since a noisy baseline signal can confound interpretation of all values. The clinical practice led to lower sample sizes for the RMT, RC, CSP and DTI values as not all patients had data for all measurements.

This is a mono-centric observational trial with a limited number of patients in a relatively new field and therefore more a proof-of-concept study. Our exploratory results should be investigated and confirmed in future studies.

## Conclusion

We identified a disturbed RC ratio as a specific feature associated with long-term motor impairments after surgery, whereas RMT and CSP indicated short-term deficits.

While the combination of different TMS-derived markers did not improve the risk-stratification overall, the addition of further TMS-derived neurophysiological parameters in the high-risk subgroup of patients with short tumor-tract distance indicates potential for enhanced individualization of preoperative risk assessment.

## Data Availability

The data supporting the findings of this study are not openly accessible due to the absence of patient consent for public data sharing. Researchers interested in accessing the data are invited to contact us directly to inquire about potential data sharing options that comply with GDPR requirements. We will assess each request individually to ensure the privacy and confidentiality of the participants are maintained.

## Abbreviations

nTMS: Navigated transcranial magnetic stimulation
RMT: Resting motor threshold
RC: Recruitment curve
CSP: Cortical silent period
TTD: Tumor tract distance
MRCS: Medical Research Council Scale
CST: Corticospinal tract
KPS: Karnofsky Performance Scale;
POD: Postoperative day
POM: Postoperative month
MPrage: 3D magnetization-prepared rapid gradient-echo
FLAIR: Fluid-attenuated inversion recovery sequence
IQR: Interquartile range
